# Retraction Note: Facile Synthesis of Mesoporous α-Fe_2_O_3_@g-C_3_N_4_-NCs for Efficient Bifunctional Electro-catalytic Activity (OER/ORR)

**DOI:** 10.1038/s41598-025-18789-y

**Published:** 2025-09-09

**Authors:** Oosamah Alduhaish, Mohd Ubaidullah, Abdullah M. Al-Enizi, Norah Alhokbany, Saad M. Alshehri, Jahangeer Ahmed

**Affiliations:** Department of Chemistry, College of Science, King Saud University, Riyadh, 11451 Saudi Arabia

Retraction of: *Scientific Reports* 10.1038/s41598-019-50780-2, published online 02 October 2019

The Editors have retracted this Article.

After publication, concerns were raised regarding the physical validity of the data presented in Figure 4. The Authors provided some source data but this was insufficient to address the concerns raised. The Editors have lost confidence in the data and conclusions of this Article.

All Authors disagree with this retraction.

